# Health and health service usage outcomes of case management for patients with long-term conditions: a review of reviews

**DOI:** 10.1017/S1463423620000080

**Published:** 2020-08-03

**Authors:** Alessandra Buja, Paolo Francesconi, Irene Bellini, Valentina Barletta, Giovanni Girardi, Mario Braga, Mimma Cosentino, Mariagrazia Marvulli, Vincenzo Baldo, Gianfranco Damiani

**Affiliations:** 1 Department of Cardiologic, Vascular, and Thoracic Sciences, and Public Health, University of Padua, Padova, Italy; 2 Toscana Regional Healthcare Agency, Florence, Italy; 3 School of Specialization in Hygiene, Preventive Medicine and Public Health, University of Padua, Padova, Italy; 4 National Health Agency, Rome, Italy; 5 Fondazione Policlinico Universitario A Gemelli IRCCS, Roma – Università Cattolica del Sacro Cuore, Rome, Italy

**Keywords:** case management, healthcare, long-term conditions, review of reviews

## Abstract

**Objective:**

There have been plenty of articles published in recent decades on patient care in the form of case management (CM), but conclusions regarding health outcomes and costs have often been discordant. The objective of this study was to examine previous systematic reviews and meta-analyses with a view to assessing and pooling the overwhelming amount of data available on CM-based health outcomes and resource usage.

**Methods:**

We conducted a review of reviews of secondary studies (meta-analyses and systematic reviews) addressing the effectiveness of CM compared with usual care (or other organizational models) in adult (18+) with long-term conditions. PubMed, the Cochrane Database of Systematic Reviews, and the Database of Abstracts of Reviews of Effects (DARE) were searched from 2000 to the end of December 2017. The outcomes of interest are related to process of care, health measures, and resource usage.

**Results:**

Twenty-two articles were ultimately considered: 4 meta-analyses and 18 systematic reviews. There is strong evidence of CM increasing adherence to treatment guidelines and improving patient satisfaction, but none of the secondary studies considered demonstrated any effect on patient survival. Based on the available literature, there is contrasting evidence regarding all the other health outcomes, such as quality of life (QOL), clinical outcomes, and functional status. Good-quality secondary studies consistently found nothing to indicate that CM prompts any reduction in the use of hospital resources.

**Conclusion:**

The source of variability in the literature on the consistency of the evidence for most outcomes is unclear. It may stem from the heterogeneity of CM programs in terms of what their intervention entails, the populations targeted, and the tools used to measure the results. That said, there was consistently strong evidence of CM being associated with a greater adherence to treatment guidelines and higher patient satisfaction, but not with a longer survival or better use of hospital resources.

## Introduction

The greatest challenge that health systems globally face in the 21st century concerns the gradual aging of the population, or rather the increasing burden of long-term conditions requiring ongoing management over a period of years or decades, and posing a great variety of health problems (Nolte and McKee, 2008: 1). A strategic vision is needed, coupled with the ability to mobilize and deliver appropriate resources to patients with long-term conditions in the community, so that healthcare professionals can provide accessible, safe, well-coordinated, cost-effective, high-quality care (Clarke *et al*., 2017). In particular, case management (CM) is a method designed to provide intensive, personally tailored care to meet the needs of patients with multiple chronic conditions who are at greatest risk of needing hospitalization and responsible for the highest costs (Hutt *et al.*, 2004). This CM approach was defined by the Case Management Society of America as ‘a collaborative process of assessment, planning, facilitation, care coordination, evaluation, and advocacy for options and services to meet an individualʼs and familyʼs comprehensive health needs through communication and available resources to promote quality, cost-effective outcomes’ (Case Management Society of America, 2007). However, there are multiple components and variations of CM depending on the context and client population, in fact there are complex interdependent and dependent factors influencing what CM interventions are done, when, with whom, and in what context (Lukersmith *et al.*, 2016).

Numerous systematic reviews and meta-analyses addressing the efficacy/effectiveness of CM have been published in recent years, often with discordant conclusions as regards both health outcomes and healthcare resource consumption (Hallberg and Kristensson, 2004). For example, Stokes *et al*. (2015) found insignificant differences in total costs, mortality, and service usage when they examined community-based CM for adults with longstanding clinical conditions and ‘at risk’ of hospitalization. In contrast, Hickam *et al*. (2013) reported that CM programs can help frail elderly people to avoid or contain functional loss, improve quality of life (QOL), and remain independent, and that CM programs also have the potential to forestall hospitalizations, emergency department (ED) visits, and skilled nursing facility use. Such incongruent evidence on the impact of CM may be a serious issue from the point of view of policy-makers and health system planners. The aim of the present study was to conduct a review of reviews of existing systematic reviews and meta-analyses on CM to pool and examine the abundance of research available on CM-related health outcomes and resource usage.

## Methods

### Review of reviews methods

Review of reviews systematically seek, organize, and assess existing evidence from multiple systematic reviews and/or meta-analyses on all the health outcomes associated with a given exposure.

We conducted a review of secondary studies (meta-analyses or systematic reviews, excluding reviews that were not systematic) on the efficacy/effectiveness of CM schemes for patients with longstanding conditions on multiple health outcomes and/or health service usage outcomes.

### Literature search

PubMed, the Cochrane Database of Systematic Reviews (CDSR), and the Database of Abstracts of Reviews of Effects (DARE) were searched from 2000 up to the end of December 2017. The time window examined is much wider, however, since our search strategy identified reviews that included papers published in earlier years. The detailed search strategy used for PubMed, and also adapted for the CDSR and DARE, was as follows: *(case managed[Title] OR case management[Title] OR case management,[Title] OR case manager[Title] OR case managers[Title] OR case managing[Title]) AND (review[ptyp] AND (‘2000/01/01’[PDAT] : ‘2017/12/31’[PDAT])*. A manual search was also conducted in the reference lists of relevant articles and in the gray literature to identify additional papers. Studies were selected in two stages. First, the titles and abstracts of the studies identified were screened in full by two authors (AB and GG). Then, the full texts of the selected papers were retrieved and independently reviewed against the inclusion/exclusion criteria by the same two authors (AB and GG), who then discussed any papers on which they disagreed to arrive at a consensus of opinion.

### Eligibility criteria and data extraction

Reviews were eligible for inclusion if they were written in English or Italian, were published between 2000 and 2017, and met the following criteria:

– Population: adults (18+) with long-term conditions;
– Intervention: CM. Only reviews in which CM had been analyzed separately from other organizational models were considered. Reviews addressing interventions for a specific disorder (e.g., CM for people with dementia) were excluded to avoid the overall analysis being excessively influenced by those long-term conditions with the highest incidence or most often studied;
– Comparison: usual care or other specific organizational models;
– Outcome: care processes, health measures, and healthcare resource usage.

Both quantitative and qualitative systematic reviews were considered. Finally, the selected studies were appraised in terms of their adherence to the PRISMA checklist (Moher *et al*., 2009), which consists of 27 items, each scored as being ‘met’ or ‘not met’, or ‘not applicable’. The quality of the reviews was classified as high (for PRISMA scores of 80% or more), intermediate (from 60% to 79%), or low (less than 60%).

### Data extraction

A data extraction form was prepared using Microsoft Excel. Descriptive data were extracted by one author (GG) and verified by the other (AB). The data collection included:

– general characteristics of the review: title, first author, journal, year, type (systematic review or meta-analysis; when a study was both a systematic review and a meta-analysis at the same time, it was tagged as a meta-analysis);
– methodological features: databases searched, number, type and data range of studies included, definition of CM, definition of comparison, objective, population, setting, and inclusion/exclusion criteria;
– results: review findings grouped by type of outcome as listed above:
  ○ process of care: adherence to treatment guidelines (i.e., the degree to which the prescribed therapy complied with the guidelines) and patient compliance;
  ○ health measures: clinical outcomes, depression and mental health symptoms, QOL, functional status, patient satisfaction, and survival;
  ○ resource usage: primary care usage, ED visits, hospital admissions, length of stay, and total costs of care.
– conclusions and recommendations for practice.

### Summary of evidence

The following criteria were applied to summarize the evidence:

– a high consistency of the evidence was assumed if most of the secondary studies found CM effective or ineffective for a given outcome;
– a low consistency of the evidence was acknowledged if the number of the studies claiming that CM was effective on a given outcome was comparable with the number of studies indicating that this was not.

## Results

**Figure 1.**
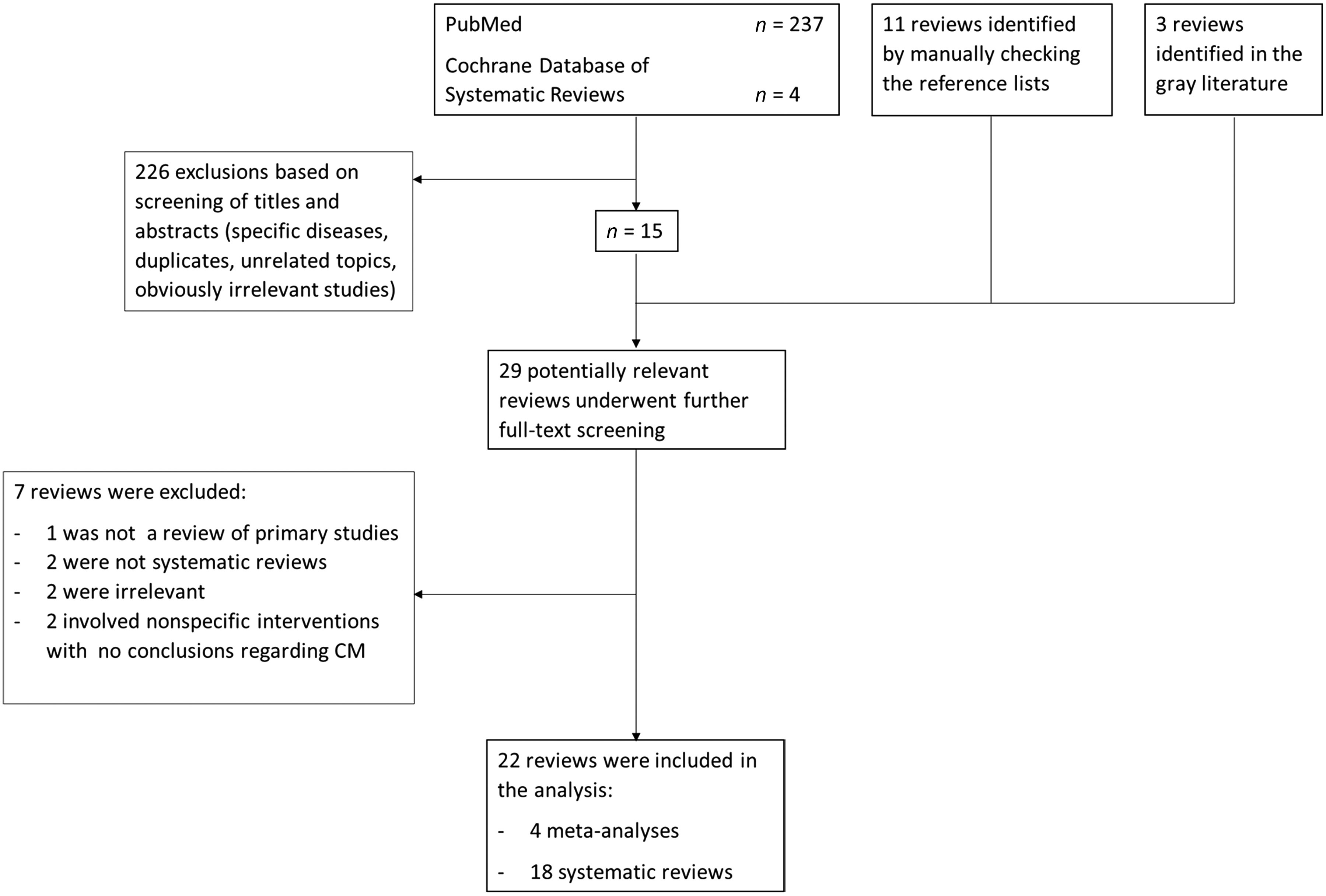
Flow diagram.

Figure 1 shows the flow diagram with the number of studies included at each stage of the selection process. The database search generated 241 potentially relevant articles. After screening the titles/abstracts and checking the references and gray literature, 29 articles were selected for full-text analysis and assessed vis-à-vis the eligibility criteria (see Figure 1 for the reasons for exclusion). This led to 22 articles being included in the present study: 4 meta-analyses and 18 systematic reviews. The review quality assessment rated the 4 meta-analysis as high quality, while the 18 systematic reviews were rated as high quality in 6 cases, intermediate-quality in 5, and low quality in 7.

An overall description of the reviews considered is available in Table 1 (while the adherence of the studies to the PRISMA checklist is reported in the Appendix, Table I in Supplementary material).

**Table 1.** Included studies

Article	CM definition	Objective	Eligibility criteria and included studies
Huntley *et al*. (2013) (Family Practice) Is case management effective in reducing the risk of unplanned hospital admissions for older people? A systematic review and meta-analysis	A collaborative process of assessment, planning, facilitation, care coordination, evaluation, and advocacy for options and services to meet an individualʼs and familyʼs comprehensive health needs through communication and available resources to promote quality cost-effective outcomes.	The aim of this study was to conduct a systematic review of the randomized controlled trial (RCT) evidence for the effectiveness of case management in reducing unplanned hospital admissions for older people.	Inclusion criteria were as follows: RCTs of CM initiated either in or after discharge from acute care hospitals, including the emergency department (ED), or in the community for the older population, in which one of the outcomes was number of unplanned hospital admissions or readmissions; that were either published in English or had an English abstract; and that were carried out in an Organization for Economic Co-operation and Development (OECD) country. This last criterion was chosen so that the results could be broadly applicable to the UK and other similar health systems. 11 RCTs from 1992 to 2011.
Kim and Soeken (2005) (Nursing Research) A meta-analysis of the effect of hospital-based case management on hospital length-of-stay and readmission	Hospital-based CM is defined as a dynamic system of care involving construction of interdisciplinary protocols, continual monitoring, and facilitation of a treatment plan.	The purpose of this study was to investigate the effect of hospital-based CM as compared with usual care on length of hospital stay and readmission rate by using a meta-analytic method. The research question was, ‘Is case management effective in reducing the hospital stay of inpatients and the readmission rate?’	Inclusion criteria were as follows: (a) sample included adults aged 18 years and more; (b) intervention was hospital-based CM for inpatients; (c) the design was randomized experimental; (d) information was provided regarding the difference in length of stay or readmission rate as an outcome measure; (e) the number of participants in the study groups was reported. Studies in which patients were mentally ill or received outpatient services were excluded. Studies implementing hospital-to-community-based or community-based CM were also excluded. 12 studies from 1992 to 2003.
Smith *et al*. (2016) (Cochrane Database of Systematic Reviews) Interventions for improving outcomes in patients with multimorbidity in primary care and community settings	The explicit allocation of tasks coordination to an appointed individual or group, postulating that the function of coordination is so important and specialized that responsibility for carrying it out needs to be explicitly allocated.	To determine the effectiveness of health-service or patient-oriented interventions designed to improve outcomes in people with multimorbidity in primary care and community settings. Multimorbidity was defined as two or more chronic conditions in the same individual.	The review included studies where CM was employed but only if it was specifically directed towards individuals identified as having multimorbidity. It included any type of intervention that was specifically directed towards a group of people defined as having multimorbidity. Only interventions based in primary care and community settings were included. Interventions included care delivered by family doctors, nurses, or other primary care professionals. Studies where multimorbidity was assumed to be the norm on the basis of individuals’ age were excluded as the interventions were not being targeted specifically at multimorbidity and its recognized challenges. This included studies where interventions were directed at communities of people based on location or age of participants in which participants could be presumed to have multimorbidity on the basis of their age or residence in a nursing home, but interventions were not designed to specifically target multimorbidity. The authors identified 18 RCTs (from 1999 to 2015) examining a range of complex interventions for people with multimorbidity. Nine studies focused on defined comorbid conditions with an emphasis on depression, diabetes, and cardiovascular disease. The remaining studies focused on multimorbidity, generally in older people.
Stokes *et al*. (2015) (PLoS One) Effectiveness of case management for ‘at risk’ patients in primary care: a systematic review and meta-analysis	A collaborative process of assessment, planning, facilitation, care coordination, evaluation, and advocacy for options and services to meet an individualʼs and familyʼs comprehensive health needs through communication and available resources to promote quality, cost-effective outcomes.	1. To synthesize the evidence for the effectiveness of case management in primary care for ‘at risk’ patients. 2. To explore whether the effectiveness of case management in primary care is moderated by the particular model of case management implemented, context, and study design.	Studies were included in this review if they met the following criteria: Population: adults (18+) with long-term condition(s) (while prevalence of multimorbidity (i.e., ‘complex’ cases) is highest in the elderly, the absolute numbers affected are greater in people under 65). Intervention: adopting methods to identify ‘at-risk’ patients suitable for CM, with the aim of preventing acute exacerbations of symptoms, and/or secondary care utilization among those at higher risk; CM, including all of the following activities: case-finding; assessment; care planning; care co-ordination; regular review, monitoring, and adaptation of the care plan; primary care/community-based management (regardless of where the case was first identified); Comparison: usual care or no CM; Outcome categories: Health – self-assessed health status, mortality; Cost – total cost of care, healthcare utilization (primary and nonspecialist care and secondary care separately), and; Satisfaction – patient satisfaction; Study design: Quantitative empirical research, meeting Cochrane Effective Practice and Organization of Care (EPOC) Group study design criteria: RCTs, non-RCTs (nRCTs), controlled before and after studies (CBA), and interrupted time series (ITS). Exclusion criteria: Case management solely targeting care for patients with mental health problems, although mental health conditions could be included where they were comorbidities alongside long-term physical conditions; Hospital discharge planning (short-term management to facilitate the transition from hospital to home; Non-English-language papers and gray literature. 36 studies (from 1994 to 2013).
Althaus *et al*. (2011) (Annals of Emergency Medicine) Effectiveness of interventions targeting frequent users of EDs: a systematic review	The coordination of health services on behalf of the patient by multidisciplinary teams composed of nurses, social workers, and physicians. Coordination tasks were allocated to a case manager, who guided the patient through the care process and provided social support. The locus of intervention was generally not limited to the hospital, often extending to the community. The teamʼs availability was limited to weekdays and during the daytime.	The purpose of this systematic review was to critically evaluate experimental and observational studies describing interventions targeting frequent users of hospital EDs	There were RCTs, non-RCTs, ITS studies, and controlled and uncontrolled before-and-after studies assessing interventions targeting adult (from 16 years of age) frequent users of hospital EDs. At least 1 outcome measure had to reportedly meet the inclusion criteria. The primary outcome was ED use, and the secondary outcomes were costs or cost-effectiveness analyses. Other outcomes were clinical outcomes, social outcomes, healthcare use (other than ED), and patient and staff satisfaction. No language or publication date restrictions were imposed. Studies that targeted only specific patient subgroups were excluded to increase homogeneity and comparativeness between studies, and because the authors focused on interventions for patients selected on a single, simple inclusion criterion, namely, the frequency of ED use. 11 studies (from 1985 to 2009): 3 RCTs, 1 controlled before and after, 2 controlled, and 6 uncontrolled before-and-after studies
Hickam *et al*. (2013) (Agency for Health Care Research and Quality) Outpatient Case Management for Adults with Medical Illness and Complex Care Needs	A process in which a person (alone or in conjunction with a team) manages multiple aspects of a patientʼs care. Key components of CM include planning and assessment, coordination of services, patient education, and clinical monitoring.	This report summarizes the existing evidence addressing the following key questions: Key Question 1: In adults with chronic medical illness and complex care needs, is case management effective in improving: (a) patient-centered outcomes, including mortality, quality of life, disease-specific health outcomes, avoidance of nursing home placement, and patient satisfaction with care? (b) quality of care, as indicated by disease-specific process measures, receipt of recommended healthcare services, adherence to therapy, missed appointments, patient self-management, and changes in health behavior? (c) resource utilization, including overall financial cost, hospitalization rates, days in hospital, ED use, and number of clinic visits (including primary care and other provider visits)? Key Question 2: Does the effectiveness of case management differ according to patients’ characteristics, including but not limited to: particular medical conditions, number or type of comorbidities, patients’ age and socioeconomic status, social support, and/or level of formally assessed health risk? Key Question 3: Does the effectiveness of case management differ according to intervention characteristics, including but not limited to: practice or healthcare system setting; case managers’ experience, training, or skills; case management intensity, duration, and integration with other care providers; and the specific functions performed by case managers? The analytical framework depicts the Key Questions in the context of the populations, intervention, and outcomes considered in the review.	Populations of interest A main criterion in choosing studies for inclusion was the existence of complex care needs. The studies included sometimes addressed populations in which psychiatric problems, such as depression or dementia, were important comorbid conditions. Studies in which the primary clinical problem was a psychiatric disorder (other than dementia) and in which CM was used primarily to manage mental illness or a substance abuse disorder were excluded. Interventions Studies in which the case manager was a licensed independent practitioner, such as a primary care physician, a geriatrician, or a nurse practitioner, were excluded. This is because such CM is part of the primary medical care provided to the patient rather than a separate clinical service. Comparators In most studies, CM is compared with usual care (i.e., care without a CM component). Usual care can vary across studies, but in most cases the comparator was the same milieu of clinical services without a distinct CM component. When a study compared two or more different types of CM, then the comparator was the alternative type of CM. Timing A level of longitudinal engagement with patients was a criterion for study inclusion. Studies that provided CM for only short durations (30 days or less) were excluded. This led to the exclusion of many studies that evaluated short-term post-hospitalization programs (often termed ‘transitional care’ programs). Such programs fall into a large category of inpatient discharge planning activities that are beyond the scope of this review. Settings Only studies on the outpatient setting, including primary care, specialty care, and home care settings, were considered. No geographic al limitations were applied. Types of Studies Randomized trials and observational studies pertinent to the Key Questions were included. The observational studies included studies using nonexperimental designs such as cohort, case–control, and pre-post designs. Previously published systematic reviews were not included as part of the evidence base but were compared with the results of this review. 109 studies (from 1989 to 2011). The majority were randomized trials. The studies were sorted by patient population and were assigned to the following categories: Older adults with one or more chronic diseases (20 studies/30 articles); Frail elderly (14 studies/17 articles); Dementia (15 studies/26 articles); Congestive heart failure (12 studies/12 articles); Diabetes mellitus (12 studies/24 articles); Cancer (6 studies/8 articles); Chronic infections (HIV or tuberculosis) (15 studies/17 articles); Other medical problems (15 studies/19 articles).
Low *et al.* (2011) (BMC Health Services Research) A systematic review of different models of home and community care services for older persons	Case management was defined as interventions where a central worker provided assessment, care planning, coordination of services and ongoing follow-up.	The aim of this review was to evaluate the outcomes of case management, integrated care and consumer-directed home and community care services for older persons, including those with dementia.	Inclusion criteria were as follows: (1) written in English; (2) evaluating the delivery of case-managed, integrated, or consumer-directed home and community services using quantitative outcomes. Home and community services could include but not be limited exclusively to medical care; (3) community dwelling, with either a majority of the sample aged 65 years and over or with a subsample of persons aged 65 and over for whom results were reported separately; (4) the sample was not selected because patients had a specific medical illness, except for dementia. There were seven RCTs (three focusing on individuals with dementia), two nonrandomized trials and three observational studies with nonmatched controls comparing case-managed care to usual noncoordinated care. The publication dates of the studies included in the review ranged from 1998 to 2008.
Purdy *et al*. (2012) (National Institute for Health Research) Interventions to reduce unplanned hospital admission: a series of systematic reviews	Case management in hospital/healthcare systems is a collaborative practice model including patients, nurses, social workers, physicians, other practitioners, caregivers, and the community. The case management process encompasses communication and facilitates care along a continuum through effective resource coordination. The goals of case management include the achievement of optimal health, access to care, and appropriate utilization of resources, balanced with the patientʼs right to self-determination.	The overall aim of this systematic review was to evaluate the effectiveness and cost-effectiveness of interventions to reduce UHA (unplanned hospital admissions). The primary outcome measures of interest were reduction in risk of UHA or readmissions to a secondary care acute hospital, for any specialty or condition.	Inclusion criteria were: all controlled studies, namely RCTs, controlled clinical trials, controlled before-and-after studies, and ITS, in which one of the outcomes was number of unplanned hospital admissions or readmissions; that were either published in English or had an English abstract; that were carried out in an OECD country. This latter criterion was chosen so that the results could be broadly applicable to the UK and other similar health systems. Studies were excluded if unplanned admissions could not be separated from planned or elective admissions using data provided in the paper or by the authors. 29 controlled studies (from 1992 to 2010), namely, randomized trials (RCTs), controlled clinical trials, controlled before-and-after studies, and ITS. Of these 29 studies, 11 concerned the elderly, 6 were on heart failure, 4 were on Chronic obstructive pulmonary disease (COPD), and 7 covered a range of other conditions such as cancer, diabetes, dementia, and stroke.
Soril *et al*. (2015) (PLoS One) Reducing frequent visits to the ED: a systematic review of interventions.	Broadly defined case or care management (CM) is considered a comprehensive, interdisciplinary approach taken to assess, plan, personalize, and guide an individualʼs health services to promote improved patient and health system outcomes. A single point of contact (e.g., an individual described as either a case manager, care manager, or ED consultant) is assigned to a frequent ED user and is tasked with brokering access and guiding the patient through their customized care process, which may extend beyond the normal continuum of the ED and in-patient care, into the community.	The objective of this research was to establish the effectiveness of interventions aimed at reducing ED utilization, in comparison to usual care, for individuals who are frequent users of the ED.	Studies were included if: they reported original data; had a control group (controlled trials or prospective comparative cohort studies); were set in an ED or acute care facility; focused on a general adult frequent ED user population; and examined the impact of an intervention to reduce the ED utilization of frequent ED users. No fixed definition of frequent user was applied; any definition used in the studies considered was accepted. Studies were excluded if they did not meet the above criteria, or if they only assessed a specific demographic or clinical group of frequent users (e.g., seniors, those with asthma, migraine sufferers, homeless). 2 RCTs and 10 comparative cohort studies (from 2000 to 2014).
You *et al.* (2013) (Journal of Aging and Health) Case managed community aged care: what is the evidence for effects on service use and costs?	The Case Management Society of Australia formally defines case management as a ‘collaborative process of assessment, planning, facilitation, and advocacy for options and services to meet an individualʼs holistic needs through communication and available resources to promote quality cost-effective outcomes’.	The study provides a systematic review to summarize the effects of CMCAC (CM in community-aged care) on service use and costs, reveal the value of CMCAC interventions, and further assist stakeholders such as aged care policy makers, aged care organizations, and case managers to make informed decisions about their services.	Types of study included: RCTs and observational comparative studies that examined the effects of CMCAC on service use and/or costs. Only studies in English and published in refereed journals or publications of equivalent standard were included. No publication date restriction was imposed. Types of participant: Participants in the studies reviewed were community-dwelling frail elderly (people aged 65 and older who suffered from age-related health problems such as functional disabilities and cognitive problems. Studies involving young adults or children, or patients with single chronic diseases were excluded. Types of intervention: This review only focused on independent CM interventions specifically applied in the community aged care setting. Studies involving more than one or multifaceted identifiable core CM functions, such as assessment, care planning, care coordination, monitoring, and so on were of particular interest. Case management interventions with the following features were excluded: applied in other community care settings, such as primary care and community mental health (studies based on primary care settings where enrolled participants had dementia instead of one specific chronic disease, such as diabetes, were included); medically focused interventions (such as some disease programs) aiming to meet participants’ medical rather than holistic care needs; single or simple preventive measure (e.g., in-home visits) differing from comprehensive CM interventions; CM playing a small part in a multifaceted intervention or an integrated care delivery system/model. 21 studies (from 1985 to 2010): 16 RCTs and 5 comparative observational studies.
Eklund and Wilhelmson (2009) (Health and Social Care in the Community) Outcomes of coordinated and integrated interventions targeting frail elderly people: a systematic review of RCTs.	CM was defined as the coordination of various system components for a successful outcome. It entails the assessment of a personʼs longer-term care needs followed by appropriate recommendations for care, monitoring, and follow-up. Five core CM activities are assessment, planning, linking, monitoring, and advocacy. A cornerstone for improving care coordination is effective information transfer between different caregivers and care levels.	The aim of this study was to review RCTs on integrated and coordinated interventions targeting frail elderly people living in the community, their outcome measurements, and their effects on the client, the caregiver and healthcare utilization.	The inclusion criteria were original article; integrated intervention including CM or equivalent coordinated organization; frail elderly people (elderly defined as 65 years or older) living in the community; RCTs; in the English language, and published in refereed journals between 1997 and July 2007. The exclusion criteria were studies targeting a specific characteristic of frailty, such as a single diagnosis or symptom; articles published before 1997; trials performed in Africa, Asia and South America; no origin or authors listed; reviews and editorials. 9 RCTs (from 1998 to 2006).
Joo and Liu (2017) (International Nursing Review) Case management effectiveness in reducing hospital use: a systematic review.	CM is a ‘collaborative process of assessment, planning, facilitation, care coordination, evaluation, and advocacy for options and services to meet an individualʼs and familyʼs comprehensive health needs through communication and available resources to promote quality, cost-effective outcomes’.	The specific aim of this systematic review was to identify and then synthesize evidence from studies published within the last 10 years on the effectiveness of CM interventions for hospital use outcomes. The research question was ‘Do CM interventions affect hospital use by individuals with chronic illnesses?’	Inclusion criteria: (1) primary research that used RCT for the study design; (2) implementations of CM interventions that had the express aim of reducing hospital use by study participants with chronic illnesses. Interventions could include any follow-up care services and transitional care services defined by the Case Management Society of America as components of CM; (3) interventions that were transferred from hospital settings to communities and that focused on transitional care services; (4) studies that included populations who were diagnosed with chronic illnesses (≥18 years of age; the disease must have been listed by the Centers for Disease Control and Prevention (2016); (5) interventions that evaluated primary hospital use outcomes such as hospital readmissions, ED visits, and length of stay in hospital; (6) studies that included psychological outcomes or cost analyses; (7) studies published in English. Exclusion criteria; Studies that mixed with other interventions or implemented CM as part of the main intervention were excluded. Studies that only described protocols for future study and planned post-test outcomes with CM intervention were also excluded as irrelevant. 10 RCTs (from 2007 to 2014).
Latour *et al*. (2007) (Journal of Psychosomatic Research) Nurse-led case management for ambulatory complex patients in general health care: a systematic review.	Case management is concerned with an optimization of multidisciplinary treatment for complex patients and the integral care needs of the individual patient without focusing on only one specific illness or population (as in disease management). The criteria used to identify case management were assessment of the clientʼs needs, development of a comprehensive service plan, arrangement of service delivery, monitoring and assessment of services, evaluation, and follow-up.	The aim was to summarize evidence for the effectiveness of post-discharge nurse-led case management for complex patients by means of a systematic review.	Studies published from 1966 until June 15, 2005, were eligible for inclusion in the review; no language restrictions were applied. Studies considered for inclusion in this review focused on ambulatory patients over 18 years of age and defined as complex. Studies were excluded if they focused on only one specific disease, with less attention paid to other vulnerabilities or comorbidities (e.g., disease management protocols) or when the CM focused solely on psychiatric/mental health care. Interventions had to be implemented in an ambulatory setting. The criteria used to identify CM were assessment of the clientʼs needs, development of a comprehensive service plan, arrangement of service delivery, monitoring and assessment of services, evaluation, and follow-up. There were no limits with regard to the types of intervention. Studies were excluded if the care was only guided by chronic disease management protocols or guidelines, or if the case manager was an administrative case manager (employed by an insurance company). Studies with one or more of the following outcome measures were included: readmission, duration of hospital readmissions, ED visits, functional status, quality of life, and patient satisfaction. 10 studies (from 1993 to 2004).
Oeseburg *et al*. (2009) (Nursing Research) Effects of case management for frail older people or those with chronic illness: a systematic review	In case management, an individual or a small team is responsible for navigating the patient through a complex process in the most efficient, effective, and acceptable way.	The aim of this study was to review RCTs systematically to determine the effects of a patient advocacy case management model on service use and costs in people with a somatic chronic disease or in frail older people living in the community.	To be considered for inclusion, studies had to evaluate CM interventions. Eligible studies reported RCT on the patient advocacy CM model and evaluated service use and costs. Studies on mental healthcare or acute care, and studies applying other CM models such as hospital-based CM, interrogative CM, disease management programs, or programs for discharge follow-up were excluded. Studies focusing on children, adolescents, caregivers, substance abuse, or professional reintegration were also excluded. 8 RCTs (from 1995 to 2007).
Thomas *et al*. (2014) (Nursing Research and Practice) Examining end-of-life (EOL) case management: systematic review.	A collaborative process of assessment, planning, facilitation, care coordination, evaluation, and advocacy for options and services to meet an individualʼs and familyʼs comprehensive health needs through communication and available resources to promote quality, cost-effective outcomes	A systematic literature review with the aim of understanding what research evidence exists on EOL case management.	The search was limited to English language research articles. Although it identified some general reviews of CM, these were excluded as none focused on end-of-life case management (EOL CM). Around 380 discussion or opinion articles on EOL CM were also rejected for review. 17 studies (from 1994 to 2010).
Boult *et al*. (2009) (Journal of the American Geriatric Society) Successful models of comprehensive care for older adults with chronic conditions: evidence for the Institute of Medicineʼs ‘Retooling for an Aging America’ report.	Care management (CM) is a collaborative model that generally involves a nurse or social worker helping chronically ill patients and their families to assess problems, communicate with healthcare providers, and navigate the healthcare system. Care managers are usually employees of health insurers or capitated healthcare provider organizations.	This study sought to identify models of comprehensive care that high-quality research has shown to be capable of improving the quality, outcomes, and efficiency of care for chronically ill older persons. The considerable heterogeneity of models, target populations, and research methods precluded meta-analyses (or even systematic reviews) of the models’ positive and negative effects. Instead, the study strove to identify promising models that should be considered for replication or further study.	MEDLINE was searched for articles published in English between January 1, 1987, and May 30, 2008, that reported statistically significant positive outcomes (improvements in the quality or efficiency of care, or in patients’ quality of life, functional autonomy, or mortality) from high-quality studies of clinical models staffed primarily by healthcare professionals to provide comprehensive health care to older persons with several chronic conditions. Models were considered to be comprehensive if they addressed several health-related needs of older persons, such as care for several chronic conditions, for several aspects of one condition, or for persons receiving care from several healthcare providers. Studies of more narrowly focused models such as innovations in cataract surgery and management of single medications were excluded. Studies were considered to be of high quality if they met five criteria: strength of design (reviews, meta-analyses, or controlled trials with equivalent concurrent control groups), adequacy of sample (adequate number of representative, chronically ill participants: 65), validity of measures, reliability of data collection techniques, and rigor of data analysis. 12 RCTs and 1 QE study (from 1999 to 2007).
Chiu and Newcomer (2007) (Professional Case Management) A systematic review of nurse-assisted case management to improve hospital discharge transition outcomes for the elderly.	The interventions varied widely in scope and duration, but common elements included home visits, telephone contact, and training in self-management. Liaison and coordination with patients’ physicians and other providers was a feature in about one third of the programs.	The following questions were addressed: What are the effects of the interventions on unscheduled readmission rates in hospital in comparison with usual care? What are the effects of the interventions on ED visits in comparison with usual care? What are the effects of the interventions on the length of stay during readmission in comparison with usual care? What are the effects of the interventions on mortality rates in comparison with usual care? What are the effects of the interventions on total Medicare care expenditures in comparison with usual care?	Inclusion criteria were links to full-text, clinical trials, and randomized clinical trials. Additional references were identified from the original citations. Of 323 citations identified in this manner, 89 were excluded because they dealt with the effectiveness of specific medical and/or surgical treatments; not with discharge transitions. Another 166 were excluded because they were not related to elderly and/or hospital discharge. Also excluded were 12 articles focusing on ED uses, 17 articles related to psychiatric patients, 22 articles that were not clinical trials, and 1 article that did not measure any hard outcomes. 15 clinical trials (from 1999 to 2006).
Hallberg and Kristensson (2004) (Journal of Clinical Nursing) Preventive home care of frail older people: a review of recent case management studies	CM interventions may include a comprehensive assessment, care planning as well as information and referral, direct nursing care services and coordination and monitoring of services. It should perhaps also include self-care management, general and specific health and care education, and healthcare strategies involving the older person as well as the informal caregiver and formal caregivers if they have limited training in geriatric care.	This paper explores the empirical literature for studies of case/care management (CM) interventions for community-dwelling frail older people and especially with regard to the content of the interventions, the nurseʼs role, and the outcomes.	The search was limited to studies published in English that included an abstract and that concerned people 65 years or older. Studies focusing on a particular group of diseases, such as chronic obstructive pulmonary disease, stroke and stroke rehabilitation, dementia-related disease, or heart disease, were excluded. 26 studies (from 1980 to 2004).
Hutt *et al.* (2004) (Kingʼs Fund) Case-managing long-term conditions: what impact does it have in the treatment of older people?	Case management has been defined as the process of planning, coordinating, managing and reviewing the care of an individual. The broad aim is to develop cost-effective and efficient ways of coordinating services in order to improve quality of life. It has its roots in social care, where it was developed as a mechanism for delivering holistic individualized care, tailored to the needs of people with complex health and social care problems.	This review of published research on case management aims to: describe methods of patient selection; evaluate the impact of case management on healthcare utilization and patient health; review the reported cost-effectiveness of case management.	Inclusion criteria: – CM provided by or linked to healthcare services with or without the inclusion of social care and other services; – CM intervention lasting at least three months; – the outcomes measured included a change in use of healthcare resources (although this may not have been the main focus of the study); – studies of disease-specific models of CM if they reported both general and disease-specific service use and outcomes. Studies about mental health CM or hospital-based CM with no community/primary care component were excluded. In view of current interest in CM for older people with chronic disease and complex needs, the search was restricted to studies in which the majority of subjects were over 65. 19 studies (from 1984 to 2003): 14 RCTs, 3 non-RCTs, 2 before-and-after studies.
Joo and Huber (2013) (International Nursing Review) An integrative review of nurse-led community-based case management effectiveness	Care coordination is ‘the deliberate organization of patient care activities between two or more participants (including the patient) involved in a patientʼs care to facilitate the appropriate delivery of healthcare services’	The purpose of this integrative review was to identify and synthesize the quantitative and qualitative evidence of the effectiveness of CM.	Types of study included: – limited to years 2000–2012; – limited to publications in English; – titles reviewed – included studies looking at patient outcomes. Types of study excluded: – intervention-launching articles, reviews, case manager development articles and studies where CM was not the main intervention. 18 studies (from 2000 to 2013): 7 RCTs and 11 of other type.
Kumar and Klein (2013) (Journal of Emergency Medicine) Effectiveness of case management strategies in reducing ED visits in frequent user patient populations: a systematic review	Case management (CM) is defined as a ‘collaborative process of assessment, planning, facilitation, and advocacy for options and services to meet an individualʼs health needs through communication and available resources to promote quality cost-effective outcomes’.	The CM literature was systematically reviewed to determine the proven effectiveness of this model in the frequent ED user patient population. This review focuses on evidence of the impact of CM as an intervention in improving outcomes of frequent users of ED care. The primary outcome of interest was ED utilization, although some studies did report cost analyses and psychosocial outcomes as well.	Limits used for each search phase included publications dating from 1990 to April 2011, human subjects, age >18 years, and English language. The targeted study population was patients >18 years of age designated as frequent users of the ED without specific limitations on medical condition, reason for ED utilization, or complaint. The interventions studied had to be identified as CM interventions and the study had to report at least one outcome with this intervention. 12 studies (from 1996 to 2011), including both prospective and retrospective studies, randomized and non-RCTs, case–control studies, and pre- and post-intervention analyses using historical controls.
Lupari *et al*. (2011) (Journal of Clinical Nursing) ‘We’re just not getting it right’ – how should we provide care to the older person with multimorbid chronic conditions?	Case management was defined as a nurse providing targeted care to individual patients, which included clinical and social support, assessment, planning, implementation and monitoring or organizing care provision to prevent and/or minimize exacerbations in the individualʼs chronic condition(s).	The aim of this literature review was to appraise available research and service evaluation evidence on nurse-led case management services targeting older people with multiple chronic conditions in their own homes.	One inclusion criterion established that the studies should compare a CM intervention with usual care in the home setting. Studies would only be included if they were able to answer the question ‘Is nurse-led case management for older people with multiple chronic conditions more effective than usual care in their own homes?’. Studies were included if published in English. 8 studies (from 1996 to 2008).

Tables 2, 3, and 4 summarize the main characteristics of all the available reviews and meta-analyses.

**Table 2.** Processes of care

Outcome	Type	Author, year	Findings	Consistency of evidence
Patient compliance and adherence of treatment prescriptions to guidelines	M_A	Smith *et al*. (2016)	2/4 studies found a positive effect of CM relating to medication use and adherence, while 2/4 did not. The range of standardized effect sizes indicated minimal effects of the interventions.	High consistency of evidence of effectiveness of CM improving adherence of treatment prescriptions to guidelines. Low consistency of evidence regarding patient compliance.
	SR_A	Hickam *et al*. (2013)	1/1 study on older adults with one or more chronic diseases found no difference in self-management understanding and adherence.	
	SR_A	Low *et al.* (2011)	2/3 studies showed improvements in the management of medication. 1/3 studies reported no difference in the management of medication.	
	SR_C	Boult *et al*. (2009)	4/4 studies found positive results in a set of compliance measures (↑ use of appropriate meds; ↑ adherence to guidelines; ↑ care quality; ↑ self-care behavior).	

M_A = high-quality meta-analyses; SR_A = high-quality systematic reviews; SR_B = intermediate-quality systematic reviews; SR_C = low-quality systematic reviews.

**Table 3.** Health measures

Outcome	Type	Author, year	Findings	Evidence
Depression	M_A	Smith *et al*. (2016)	5/7 studies showed improvements in a range of depression measures. 2/7 studies found no improvements in depression outcomes. A meta-analysis of Patient Health Questionnaire depression scores and a meta-analysis of standardized mean differences (MDs) in depression scores suggested a modest effect of the intervention.	High consistency of evidence
	SR_A	Low *et al.* (2011)	2/3 studies found no difference in depression and psychological health, while 1/3 found an improvement.	
	SR_A	Hickam *et al*. (2013)	CM programs that serve patients with dementia reduce depression and strain among caregivers (strength of evidence: moderate).	
	SR_B	Eklund and Wilhelmson (2009)	Studies significantly in favor of intervention: 2/4.	
Quality of life	SR_A	Hickam *et al*. (2013)	CM improves selected cancer-related symptoms and functioning (physical, psychosocial, and emotional), but not overall quality of life or survival (8 studies). CM programs that serve patients with CHF do improve CHF-related quality of life.	Mixed consistency of evidence
	SR_A	Low *et al.* (2011)	1/2 studies found a higher quality of life among CM patients, while 1/2 found no difference.	
	SR_B	Eklund and Wilhelmson (2009)	1/3 studies were significantly in favor of the intervention.	
	SR_B	Joo and Liu (2017)	1/2 studies found no difference in quality-of-life scores between the CM group and the control group after six months of CM implementation. 1/2 studies found significant positive effects on quality-of-life for the intervention group after two years of nurse-led CM intervention.	
	SR_B	Latour *et al*., 2007	3/4 studies (1 high-quality and 2 low-quality) found no difference in quality of life between the intervention and the control groups. Only one low-quality study reported a significant difference in favor of the intervention group.	
	SR_C	Boult *et al*. (2009)	7/8 studies found positive results in a set of quality-of-life measures (less decline in SF-36 social function; ↑ control of fatigue and mastery; ↑ SF-36, ↑ social support; ↑ SF-36; ↑ Minnesota Living with Heart Failure scores).	
	SR_C	Joo and Huber (2013)	Overall, community-based CM done by nurses enhanced patients’ quality of life	
Clinical outcomes	M_A	Smith *et al*. (2016)	The MD in glycemic control between the intervention and control groups was 0.02 (95% CI − 0.21 to 0.25). The MD in blood pressure between the intervention and control groups was −3.10 (95% CI − 7.26 to 1.06).	Low consistency of evidence
	SR_A	Hickam *et al*. (2013)	1/1 study found a moderate decrease in blood pressure, glucose and cholesterol levels after the CM intervention.	
	SR_A	Low *et al.* (2011)	One study reported a reduction in pain among CM patients and another showed an improvement in physical health.	
Functional status	SR_A	Hickam *et al*. (2013)	CM programs that serve patients with one or more chronic diseases do not result in clinically important improvements in functional status (strength of evidence: high).	Low consistency of evidence
	SR_A	Low *et al.* (2011)	3/5 studies showed improvements in functional status ACTIVITIES OF DAILY LIVING/INSTRUMENTAL ACTIVITIES OF DAILY LIVING (ADL/IADL). 2/5 studies reported no difference in functional status (ADL/IADL).	
	SR_B	Eklund and Wilhelmson (2009)	4/6 studies reported no difference in functional status (ADL). 2/6 studies showed improvements in functional status (ADL).	
	SR_B	Latour *et al*., 2007	2/2 studies (1 high-quality and 1 low-quality) found no significant difference in functional status between intervention and control groups.	
	SR_C	Boult *et al*. (2009)	Weak evidence of an improved functional autonomy (1/4 studies).	
	SR_C	Hallberg and Kristensson (2004)	3/5 studies reported positive results in functional status measures (ADL, IADL).	
	SR_C	Hutt *et al.* (2004)	3/6 RCTs showed statistically significant positive results for case-managed patients compared with other patients, in terms of either less decline in functional ability or an improvement in function. 1/6 RCTs found positive results, but they were not statistically significant. 2/6 RCTs revealed no differences between control and intervention groups. Non-RCTs: one before-and-after study showed a positive effect associated with CM.	
	SR_C	Joo and Huber (2013)	1/2 studies found an improvement in ADL and IADL for the CM group after one year of follow-up, while scores on these scales deteriorated in the control group. 1/2 studies showed no improvement in ADL and IADL.	
Survival	M_A	Stokes *et al*. (2015)	No significant effect on mortality (short-term: 0.08, 95% CI −0.03 to 0.19, *I*^2^ = 63.6%, *P* = 0.001, 12 studies; long-term: 0.03, 95% CI −0.04 to 0.09, *I*^2^ = 40.0%, *P* = 0.067, 13 studies).	High consistency of evidence of CM having no effect
	SR_A	Hickam *et al*. (2013)	CM programs that serve patients with multiple chronic diseases do not reduce overall mortality (strength of evidence: high). CM does not affect mortality in frail elders (strength of evidence: low).	
	SR_B	Eklund and Wilhelmson (2009)	1/4 studies showed a reduction in the mortality risk. 3/4 studies reported no difference in the mortality risk.	
	SR_C	Boult *et al*. (2009)	4/8 studies reported positive results for mortality.	
	SR_C	Chiu and Newcomer (2007)	Most trials had comparable death rates among the intervention and control groups.	
	SR_C	Lupari *et al*. (2011)	One study reported no significant effect on mortality.	
Patient satisfaction	M_A	Stokes *et al*. (2015)	Patient satisfaction showed a statistically significant improvement in the CM group in the short-term (0.26, 95% CI 0.16 to 0.36, *I*^2^ = 0.0%, *P* = 0.465, 8 studies), which increased in the long-term (0.35, 95% CI 0.04 to 0.66, *I*^2^ = 88.3%, *P* < 0.001, 4 studies).	High consistency of evidence of CM being effective
	SR_A	Althaus *et al*. (2011)	1/1 study reported no significant difference in patient satisfaction after the intervention.	
	SR_A	Hickam *et al*. (2013)	CM programs that serve patients with one or more chronic diseases increase patients’ perceptions that their care is better coordinated and of higher quality (strength of evidence: high).	
	SR_A	Low *et al.* (2011)	One study found no difference in satisfaction with care, while another reported a higher life satisfaction among CM patients.	
	SR_B	Eklund and Wilhelmson (2009)	3/5 studies were significantly in favor of the intervention.	
	SR_B	Latour *et al*. (2007)	2/3 studies (one high-quality and one low-quality) reported a positive effect of CM on patient satisfaction. 1/3 (high-quality) studies found no significant difference between the intervention and control groups.	
	SR_B	Thomas *et al*. (2014)	3 studies found a positive effect of CM programs on client satisfaction.	
	SR_C	Hallberg and Kristensson (2004)	2/5 studies reported that the study group was more satisfied than the control group. 2/5 studies reported no effect on patient satisfaction. 1/5 studies reported a more satisfied control group.	
	SR_C	Joo and Huber (2013)	Overall, community-based CM done by nurse case managers enhanced patients’ satisfaction.	
	SR_C	Lupari *et al*. (2011)	4/4 studies reported high levels of satisfaction with nurses and their delivery of the CM intervention for complex patients.	

M_A = high-quality meta-analyses; SR_A = high-quality systematic reviews; SR_B = intermediate-quality systematic reviews; CHF = congestive heart failure; SR_C = low-quality systematic reviews; RCTs = randomized controlled trials.

**Table 4.** Resource usage

Outcome	Type	Author, year	Findings	Evidence
Primary care	M_A	Stokes *et al*. (2015)	No effect on usage of primary and nonspecialist care (short-term: −0.08, 95% CI −0.22 to 0.05, *I*^2^ = 79.2%, *P* < 0.001, 16 studies; long-term: −0.10, 95% CI −0.29 to 0.09, *I*^2^ = 78.6%, *P* < 0.001, 7 studies)	Low consistency of evidence
	SR_A	Althaus *et al*. (2011)	2/6 studies confirmed a benefit of the intervention on the use of ambulatory care. One study reported an increase in primary care (19%; *P* = 0.003) and community care engagement (52%; *P* = 0.001) Another study described a significant increase in the median number of medical outpatient visits (1; *P* = 0.01) and a significant reduction in the number of patients lacking a primary care practitioner (−74%; *P* = 0.01).	
	SR_A	Hickam *et al*. (2013)	CM does not reduce nursing home admissions for frail elderly (strength of evidence: low).	
	SR_A	Low *et al.* (2011)	2/2 studies found an increased use of community services. 4/5 studies found a reduction in the risk of nursing home admission among CM patients, while 1/5 study found no difference.	
	SR_A	You *et al.* (2013)	Moderate evidence of CMCAC interventions significantly improving clients’ use of some community care services (greater likelihood, higher intensity, higher frequency, and earlier use), delaying nursing home placement, reducing nursing home admissions, and shortening length of nursing home stays.	
	SR_B	Eklund and Wilhelmson (2009)	The use of home services showed favorable results both in the intervention group (1/5 studies) and in controls (2/5 studies). Another 2 studies found no difference in primary care usage.	
	SR_B	Oeseburg *et al*. (2009)	3/3 studies reported no difference in the number of nursing home admissions.	
	SR_C	Lupari *et al*. (2011)	One study showed a reduction in General Practitioner (GP) contacts.	
ED visits	SR_A	Althaus *et al*. (2011)	5/8 studies reported a decreased ED use. 2/8 studies reported no significant change. 1/8 studies reported an increased ED use. The magnitude of the decrease or increase was documented in 5 studies; the effect of the intervention on ED use was large in all these studies, with a decrease or increase in the mean or median number of ED visits ranging from 28% to 75%.	Low consistency of evidence
	SR_A	Low *et al.* (2011)	1/4 studies found a reduction in the risk of ED admission among CM patients. 2/4 studies found no difference. 1/4 studies found an increase in the risk of ED admission.	
	SR_A	Soril *et al*. (2015)	RCTs: 1/2 studies reported no change in the mean number of ED visits following CM; 1/2 studies reported a slight decrease in median ED visits among patients in the intervention group. Comparative cohort studies: 8/9 studies observed a decrease in the mean (between −0.66 and −37 ED visits) or median (between −2.28 and −20 ED visits) number of ED visits compared to controls or before CM; 1/9 studies reported a 2.79 median increase in ED visits post-intervention.	
	SR_B	Joo and Liu (2017)	5/6 studies reported a statistically significant reduction in the number of ED visits post-CM intervention. 1/6 studies found reductions in 30- or 90-day ED visit rates for the CM group compared with the control group, but the results were not significant.	
	SR_B	Latour *et al*. (2007)	None of the 4 studies (2 low-quality, 2 high-quality) reported a positive effect on the number of ED visits.	
	SR_B	Oeseburg *et al*. (2009)	One study reported a small but clinically relevant reduction in ED visits, while another one reported an increase in the number of ED visits.	
	SR_C	Chiu and Newcomer (2007)	3/11 studies found significant reductions in presentations to an ED.	
	SR_C	Hallberg and Kristensson (2004)	2/5 studies found fewer ED visits in the study group. 1/5 studies reported no effect on ED visits. 2/5 studies recorded more ED visits in the study group.	
	SR_C	Hutt *et al.* (2004)	3/8 studies showed significant reductions in ED attendance following CM. 5/8 studies showed increases (2 significant and 3 nonsignificant) in ED attendance.	
	SR_C	Kumar and Klein (2013)	8/11 studies reported a reduction in ED use. 2/11 studies reported no significant reduction. 1/11 studies reported an increase in ED use.	
	SR_C	Lupari *et al*., 2011	2/3 studies found a reduction in ED admissions, while 1/3 studies reported no significant difference.	
Hospital admissions	M_A	Huntley *et al*. (2013)	9/11 RCTs showed no significant benefit in terms of CM reducing unplanned hospital admissions compared with usual care. One study, which recruited >50% of electively admitted patients, showed a significant reduction in hospital readmissions. CM initiated in hospital (or on discharge) versus usual care in the older population: relative rate of readmissions = 0.71(95% CI 0.49 to 1.03) Heterogeneity: *I*^2^ = 0.08; χ^2^ = 7.13; df = 2 (*P* = 0.03); *I*^2^ = 72%. *n* studies = 3; Case management initiated in the community versus usual care in the older population: mean difference in admissions 0.05 (−0.04 to +0.15) Heterogeneity: χ^2^ = 1.44; df = 2 (*P* = 0.49); *I*^2^ = 0%. *n* studies = 3.	High consistency of evidence of CM having no effect
	M_A	Kim and Soeken (2005)	Overall OR for readmission in 10 studies: 0.87 with a 95% CI of 0.69 to 1.04. The effect size can be interpreted as a 6% decrease in readmissions for patients involved in a CM program. No evidence of heterogeneity was found among the studies (QT [total heterogeneity]= 13.24, df = 8, *P* > 0.10). The effect of CM interventions in reducing readmissions did not differ by diagnosis: heart failure: OR = 0.749 with 95% CI of 0.446 to 1.052 (4 studies); frail elders: OR = 0.971 with 95% CI of 0.754 to 1.188 (3 studies).	
	M_A	Smith *et al*. (2016)	1/5 studies reported improvements for intervention group participants across a variety of measures relating to hospital admissions. 4/5 studies found no difference in admission-related outcomes.	
	M_A	Stokes *et al*. (2015)	No effect on secondary care: short-term: 0.04, 95% CI −0.02 to 0.10, *I*^2^ = 39.6%, *P* = 0.027, 23 studies; long-term: −0.02, 95% CI −0.08 to 0.04, *I*^2^ = 22.8%, *P* = 0.194, 16 studies.	
	SR_A	Althaus *et al*. (2011)	None of the 4 studies assessing hospitalization identified significant differences.	
	SR_A	Hickam *et al*. (2013)	CM programs that serve patients with one or more chronic diseases do not reduce overall rates of hospitalization (strength of evidence: moderate). CM is more effective in reducing hospitalization rates among patients with a greater burden of disease (strength of evidence: low). CM is more effective in preventing hospitalizations when case managers have more personal contact with patients and physicians (strength of evidence: low). CM does not reduce acute hospitalizations for the frail elderly (strength of evidence: low).	
	SR_A	Low *et al.* (2011)	2/3 studies found a reduction in the risk of hospital admission among CM patients, while 1/3 studies found no difference.	
	SR_A	Purdy *et al*. (2012)	Case management initiated in hospital or on discharge versus usual care in the older population: relative rate of readmissions 0.71 (95% CI 0.49;1.03). CM initiated in hospital: 2 RCTs, one demonstrated a reduction of readmission and another no reduction. CM initiated on discharge from hospital: 3/4 RCTs showed no significant difference in unplanned hospital admissions between CM and usual care, while 1/4 showed a reduction in admissions. Case management initiated in the community versus usual care in the older population: mean difference in admissions 0.05 (95% CI −0.04;0.15). 4/5 RCTs showed no advantage of CM over usual care; one RCT showed a small, insignificant reduction in the relative rate of unplanned hospital admissions at 12 months with GP-led CM compared with usual care.	
	SR_A	You *et al.* (2013)	No evidence that CMCAC interventions can significantly influence clients’ use of hospital care.	
	SR_B	Joo and Liu (2017)	Readmission rate: 3/10 studies reported statistically significant reductions in hospital readmissions; 3/10 studies reported fewer readmissions but results were not statistically significant; 4/10 studies reported no effect on readmission rates. Total number of hospital visits for each participant: 2/2 studies found statistically significant reductions in the number of hospital visits.	
	SR_B	Latour *et al*. (2007)	4/8 studies (3 high-quality and 1 low-quality) reported a positive result in the intervention group regarding readmission rates. 4/8 studies (2 high-quality and 2 low-quality) found no significantly better outcomes associated with CM.	
	SR_B	Oeseburg *et al*. (2009)	One methodologically sound study reported a small but clinically relevant decrease in hospital admissions in the intervention group, whereas another study of lower methodological quality showed a negligible increase in hospital admissions in the experimental group.	
	SR_B	Thomas *et al*. (2014)	1/2 studies found that seniors who received EOL CM for four weeks following hospital discharge were less likely to be hospitalized in the subsequent six months, compared to a control group. 1/2 studies found that case-managed elderly persons were more likely to be hospitalized and to use other health services during the last month of life than those who did not receive EOL CM.	
	SR_C	Chiu and Newcomer (2007)	8/15 studies found statistically significant differences between treatment and comparison groups in unplanned readmissions: the intervention cases had differences of at least one-third fewer readmissions than the control cases. 7/15 studies found no significant differences.	
	SR_C	Hallberg and Kristensson (2004)	In 5/9 studies the intervention group reportedly had fewer hospital (re)admissions. 4/9 studies found no effect on hospital (re)admissions.	
	SR_C	Hutt *et al.* (2004)	5/16 studies (only two of which were RCTs) demonstrated significant reductions in admissions. 4/16 studies found reductions in admissions that did not reach statistical significance. 7/16 studies found no difference. 2/16 studies reported an insignificant increase in admissions.	
	SR_C	Joo and Huber (2013)	3/4 studies found significant reductions in hospital admissions. 1/4 found no difference between the study and control groups.	
	SR_C	Kumar and Klein (2013)	In one large RCT, CM intervention yielded only a small, insignificant reduction in hospital admission rates. 3 pre- and post-intervention studies identified no significant differences in hospital admission rates.	
	SR_C	Lupari *et al*. (2011)	3/3 studies reported a reduction in hospital admissions.	
Costs	M_A	Huntley *et al*. (2013)	5/5 RCTs reported favorable cost-outcome descriptions for CM compared with usual care.	Low consistency of evidence
	M_A	Stokes *et al*. (2015)	No significant effect was found on total costs of services (short-term: −0.00, 95% CI −0.07 to 0.06, *I*^2^ = 0.0%, *P* = 0.784, 8 studies; long-term: −0.03, 95% CI −0.16 to 0.10, *I*^2^ = 46.0%, *P* = 0.116, 5 studies)	
	SR_A	Althaus *et al*. (2011)	In one RCT, total hospital costs were similar in the CM and the usual care groups when the costs of the intervention were factored in. Compared with usual care, CM was more cost-effective because it brought an improvement in clinical and social outcomes without adding to the overall costs. In 2 before-and-after studies, the intervention produced a cost saving: the reduction in hospital costs was larger than the cost of the CM team.	
	SR_A	Hickam *et al*. (2013)	• CM programs that serve patients with one or more chronic diseases do not reduce Medicare expenditures (strength of evidence: high).	
	SR_A	Purdy *et al*. (2012)	5/5 RCTs reported favorable cost outcomes for CM compared with usual care.	
	SR_A	Soril *et al*. (2015)	2 RCTs: one reported a significantly smaller increase in the cost of care among patients exposed to the CM intervention compared to those in the control group (CM: $3116 added costs per patient versus control: $6659 added costs per patient; *P* < 0.01). The specific cost of the CM intervention was reportedly $3633 per patient. The other RCT estimated a 45% decrease in costs (statistically significant, *P* = 0.004). 4/4 comparative cohort studies reported lower hospital costs (i.e., ED and in-patient charges) per patient in the 12 months following a CM intervention.	
	SR_A	You *et al.* (2013)	No evidence indicated that CMCAC interventions could significantly influence costs.	
	SR_B	Eklund and Wilhelmson (2009)	1/4 studies showed a reduction in costs among CM clients. 3/4 studies reported no difference in costs between the study and control groups.	
	SR_B	Joo and Liu (2017)	One study reported significant reductions in the total healthcare costs for the intervention group (45% less per person, *P* = 0.004). In another study, the cost of ED services decreased for the CM intervention group vis-à-vis the control group (*P* < 0.01), but the total hospital costs showed no difference between the two groups.	
	SR_B	Oeseburg *et al*. (2009)	1/3 studies found extensive savings for the intervention group. 1/3 studies found statistically significant but practically negligible savings for the intervention group. 1/3 found a statistically insignificant increase in the costs for the intervention group.	
	SR_B	Thomas *et al*. (2014)	4/6 studies found an economic benefit. 2/6 studies did not.	
	SR_C	Boult *et al*. (2009)	1/3 studies found that CM was less expensive than usual care. 2/3 studies reported no difference in cost between CM and usual care.	
	SR_C	Chiu and Newcomer (2007)	6/6 studies (3 of which addressed heart failure) showed lower expenditures in the intervention group. In most studies, comparisons were for hospital expenditures, but a few included community service expenditures and the cost of the intervention too.	
	SR_C	Joo and Huber (2013)	According to these studies, community-based CM improved cost-effectiveness.	
	SR_C	Kumar and Klein (2013)	3/4 studies (3 before-and-after studies) noted a significant reduction in ED costs among patients enrolled in CM interventions. 1/4 studies cited an insignificant reduction in ED-related costs.	
Length of stay (LOS) in hospital	M_A	Kim and Soeken (2005)	CM intervention was not effective in reducing LOS: the overall average-weighted effect size (AWES) for 10 studies was 0.094 (*Z* = 1.46, *P* =.07) based on *n* = 2666 with a 95% CI of –0.032 to 0.220. CM was: effective for heart failure: AWES = 0.241 (*Z* = 2.059, *P* = .02) with a 95% CI of 0.012 to 0.470; not effective for stroke: AWES = –0.226 (*Z* = –1.404, *P* = .08) with a 95% CI of –0.542 to 0.089; not effective for frail elderly: AWES = 0.126 (*Z* = 1.242, *P* = 0.11) with a 95% CI of –0.073 to 0.324.	Low consistency of evidence
	SR_A	Low *et al.* (2011)	2/3 studies found no difference in LOS, while 1/3 studies found a reduction.	
	SR_A	Purdy *et al*. (2012)	3/6 RCTs identified a significantly reduced LOS with CM compared to usual care. 3/6 RCTs did not provide this information (although one study showed a significant increase in the number of days elapsing before the first readmission).	
	SR_A	You *et al.* (2013)	No evidence of CMCAC interventions being able to significantly influence clients’ use of hospital care.	
	SR_B	Eklund and Wilhelmson (2009)	4/7 studies showed a reduction in the days spent in hospitals/institutions between CM patients and non-CM patients. 3/7 studies reported no difference in the days spent in hospitals/institutions between the two groups.	
	SR_B	Joo and Liu (2017)	1/3 studies reported a significant (*P* = 0.005) difference one year after intervention (with 29% fewer days spent in hospital). 2/3 studies found reductions in LOS, but they were not statistically significant.	
	SR_B	Latour *et al*. (2007)	4/6 studies (2 high-quality and 2 low-quality) showed a positive result in the intervention group. 2/6 studies (both of high quality) reported no significant differences compared to the control group.	
	SR_B	Oeseburg *et al*. (2009)	Only 1/5 studies reported a negligible reduction in the number of days spent in a hospital per year in the intervention group.	
	SR_C	Chiu and Newcomer (2007)	7/9 studies showed statistically significant reductions in the number of hospital readmission days or LOS. The differences in mean LOS were of the order of at least two days (and up to four days) and reflected a reduction of at least one-third in the number of days.	
	SR_C	Hallberg and Kristensson (2004)	In 5/7 studies, the intervention group was reported to have a shorter LOS. 2/7 studies found no effect on number of days in hospital.	
	SR_C	Hutt *et al.* (2004)	2/10 RCTs found a reduction in LOS. 8/10 RCTs found no statistically significant effects on overall LOS associated with CM. 2/6 non-RCTs demonstrated a significant difference in mean LOS. 4/6 non-RCTs did not find any significant differences associated with CM.	
	SR_C	Joo and Huber (2013)	3/3 studies showed positive results for LOS.	
	SR_C	Kumar and Klein (2013)	No significant reduction in medical inpatient days or psychiatric inpatient days associated with CM intervention.	
	SR_C	Lupari *et al*. (2011)	1/3 studies found a reduction in LOS, while 2/3 reported no statistically significant difference.	

M_A = high-quality meta-analyses; SR_A = high-quality systematic reviews; CMCAC = case management in community-aged care; SR_B = intermediate-quality systematic reviews; SR_C = low-quality systematic reviews; ED = emergency department; RCTs = randomized controlled trials; EOL CM = end-of-life care management.

Concerning the process of care (Table 2), secondary sources assessing the adherence of prescriptions to treatment guidelines agreed that CM was more effective than usual care.

The findings were mixed as regards patient compliance, however, with comparable proportions of the reviews (two on each side) finding or failing to find benefits of CM on this parameter. Cochrane meta-analysis (Smith *et al*., 2016) assessed the effectiveness of interventions in patients with multimorbidity in primary care and community settings, including 18 generally well-designed randomized controlled trials (RCTs). Analyzing the only four organizational studies reporting measures relating to the use of medication and patient adherence, it was found higher proportions of patient adherence among participants involved in the intervention, which were in the range of 10% to 40% in absolute terms, but all studies had small effect sizes.

As for service responsiveness to patients’ expectations (Table 3), there was a strong concordance among the studies (7 out of 10) regarding the effectiveness of CM in improving patient satisfaction. In particular, Stokes *et al*. (2015) found a statistically significant beneficial effect on patient satisfaction in the CM group in the short term, which increased in the longer term. Hickam *et al*. (2013) reported that CM interventions were generally associated with improved patient (and caregiver) satisfaction, although satisfaction with the CM itself varied across interventions. Studies measuring patient satisfaction typically reported overall satisfaction with care, rather than satisfaction in specific domains. Some studies found that CM improved patients’ perceptions of coordination among healthcare providers.

Regarding health outcomes, none of the secondary studies found any effect of CM on either short- or long-term survival.

On the issue of depression, the secondary studies identified highly consistent results. In a meta-analysis of 18 RCTs examining a range of complex interventions for people with multimorbidity, Smith *et al*. (2016) found that mental health outcomes improved, with modest reductions in mean depression scores for the comorbidity studies targeting participants with depression. Hickam *et al*. (2013) found moderate evidence of CM programs for patients with dementia reducing their depression and the strain on caregivers.

Hickam *et al*. (2013) also found that CM interventions produced mixed results in terms of improving patients’ QOL. On the whole, CM sometimes succeeded in improving aspects of QOL targeted directly by the interventions. For instance, CM improved caregiver stress among individuals caring for patients with dementia and also raised congestive heart failure (CHF)-related QOL among patients with this chronic heart condition. The measures used to assess QOL varied across studies, and any improvements in QOL achieved by CM were either small or of unclear clinical significance. CM was less successful in improving overall QOL, as indicated by global measures not specific to a particular condition. When Joo *et al*. (2013) investigated the effectiveness of nurse-led, community-based CM, they found that it generally enhanced patients’ QOL. Boult *et al*. (2009) likewise found that 7/8 studies produced positive results for a set of QOL measures (less decline in SF-36 social function; greater control of fatigue and improved mastery; an increase in social support).

A low consistency among studies emerged regarding the effectiveness of CM on patients’ functional status. Hickam *et al*. (2013) found that CM programs serving patients with one or more chronic diseases do not result in clinically important improvements in their functional status (three studies). Boult *et al*. (2009) also said that CM studies produced weak evidence of patients achieving a greater functional autonomy. When Hutt *et al*. (2004) analyzed the effectiveness of CM on long-term conditions, they found 6 RCTs that had considered functional ability as an outcome: 3 of them reported better results for patients who were involved in CM schemes than for those who were not, in terms of either a more limited decline in functional ability or an improvement in function.

Based on the available literature, contrasting data emerged regarding clinical outcomes. In particular, Hickam *et al*. (2013) found only one observational study with a pre-post design that had examined changes in physiological measures after three months of CM. Blood pressure and glucose and cholesterol levels showed a moderate decrease compared with pre-CM values. Smith *et al*. (2016) wrote that five studies had reported on six measures of glycemic control with a mean difference (MD) equal to 0.02 (95% CI − 0.21 to 0.25), and four studies reported on systolic blood pressure (SBP), describing a MD of −3.10 (95% CI − 7.26 to 1.06).

As for resource usage (Table 4), secondary studies (4 meta-analyses and 5 systematic reviews) consistently returned a lack of evidence of CM producing any reduction in hospital admissions. For example, Huntley *et al*. (2013) found that 9 of 11 studies (including RCTs) had found no decline in unplanned hospital admissions with CM compared with usual care. Stokes *et al*. (2015) also reported no effect on secondary care in either the short term (0.04, 95% CI −0.02 to 0.10, *I*^2^ = 39.6%, *P* = 0.027) or the longer term (−0.02, 95% CI −0.08 to 0.04, *I*^2^ = 22.8%, *P* = 0.194, 16 studies).

When overall expenditure was considered, the consistency was low once again. Hickam *et al*. (2013) found that studies examining the impact of CM for adults with chronic medical illnesses and complex care needs on the overall cost of care had identified no significant differences between patients receiving CM and controls. Although the cost of CM programs was often modest relative to the overall costs of patients with high levels of health service usage, the effect of CM in reducing said usage was minimal. On the other hand, when Soril *et al*. (2015) looked at CM interventions to reduce frequent visits to the ED, they found two RCTs that had specifically assessed the costs of CM programs from a health system perspective. One identified an increase in the cost of care for all participants over a 12-month follow-up, but this cost increase was significantly smaller for the patients exposed to the CM intervention than for those in the control group. The other study estimated a (statistically significant) 45% reduction in the cost of care for the CM intervention group.

## Discussion

This review of reviews not only provides strong evidence of CM improving adherence to treatment guidelines and patient satisfaction but also shows that CM has no impact on patient survival or hospitalization rates. Inconsistent evidence emerged on whether or not the other outcomes addressed are improved by CM.

Going into more detail, our review of reviews seems to show that CM ameliorates care processes. In particular, all the secondary studies considered consistently demonstrated that CM improved the adherence of prescriptions to the guidelines. In fact, CM interventions often employed clinical pathways to arrange personalized treatment plans (Kim and Soeken, 2015), making it easier for prescriptions to reflect evidence-based care (Chawla *et al*., 2016). As for patients’ compliance to their drug prescriptions, people with multimorbidity frequently have specific difficulties relating to polypharmacy and the management of complex medical treatment regimes, so CM interventions to adjust their medication may facilitate their compliance (Brown and Bussell, 2011). Our review of reviews found inconsistent evidence of CM succeeding in improving this outcome, however.

Concerning health measures, all the secondary studies confirmed the effectiveness of CM in enhancing patient satisfaction. This is in line with other reports that CM implemented by nurses improves patient-centered care, empowering patients to manage their own health, control their symptoms, and improve their QOL and helping them to stay independent for longer (Dorr *et al*., 2006). Developing patients’ self-management skills could also help to reinforce their satisfaction with their care.

On the other hand, all the secondary studies that we analyzed were unable to demonstrate the effectiveness of CM in improving overall survival. Several authors tried to explain the reasons for the limited impact of CM on this parameter, most of them arguing that the intervention period may have been too short to make a difference, or that the follow-up time was too short to detect a difference (Eklund and Wilhelmson, 2009). Starfield suggested instead that the limited influence of CM may be due to excellent usual care. In other words, CM may simply be replacing some of the functions of a well-coordinated, person-centered primary care (Starfield *et al.*, 2005). Stokes *et al*. (2015) tested this hypothesis, confirming that the effects of CM on overall survival are greater when it is delivered in contexts where routine primary care services are less well developed, whereas the capacity of CM to improve overall survival is minimal in settings with strong primary care services. Other subgroup analyses performed on the same studies indicated that CM by a multidisciplinary team (as opposed to a single case manager) that included a social worker improved short-term (but not long-term) mortality. These findings are consistent with the wider literature advocating the use of a multidisciplinary team to manage patients with long-term conditions successfully (Wagner, 2000) and promote a stronger integration of health and social care (Valentjin *et al*., 2013).

As for the impact of CM on healthcare expenditures, our review of reviews cannot sustain the efficacy of CM in containing acute hospitalizations, especially in the light of the findings of the high-quality systemic reviews and meta-analyses we considered. Here again, Stokes conducted subgroup analyses to see if certain characteristics of CM can improve this outcome, confirming that CM performed significantly better vis-à-vis the usage of secondary care in countries less oriented toward primary care delivery by multidisciplinary teams, and with the involvement of a social worker (Stokes *et al*., 2015). Both Huntley and Hickam also suggested that the effectiveness of an intervention relates to the profile of the population recruited, and they both concluded that CM is more effective in reducing hospitalization rates among patients with a greater burden of disease (Hickam *et al*., 2013; Huntley *et al*., 2013). This last issue appears to be strongly influenced by the type of risk tool used to target patients most likely to benefit from CM. In fact, Stokes *et al*. (2015) demonstrated a greater efficacy of CM in reducing secondary care service usage in the short term for programs that adopted a risk modeling approach rather than relying on clinical judgment, although the difference was not statistically significant.

This review of reviews has some limitations to bear in mind. The most significant concerns the lack of any indication of the overlap between the primary studies included in each of the secondary analyses. For example, some studies may have been included in multiple analyses, making their findings more influential because they were counted more than once (Aromataris *et al*., 2015). Theoretically, this means that the less recent studies acquire more weight as they are more likely to have been included in earlier and later analyses, whereas the most recent studies can clearly have only been the object of one secondary analysis since their publication.

Another important limitation of our review of reviews concerns the marked differences between the characteristics of the primary studies on CM. Most of the systematic reviews and meta-analyses had already highlighted this issue, but a review of reviews pooling secondary analyses together naturally makes this issue even more prominent, complicating the comparison between the studies and hampering the opportunity to make conclusive statements. The main variations concerned sample size, population characteristics, patient identification and assessment, measurements of outcome, content and duration of the CM intervention, context-related barriers and facilitators within organizations, length of follow-up, and outcomes. Information on the content of the intervention was often rather scarce, as was the information provided on the care given to the control groups.

## Implications for research

Our review of reviews found a low consistency of the evidence of the impact of CM for most of the outcomes considered. It is hard to say whether this stems from the intrinsic variability of the outcome measures or, more plausibly, from the heterogeneity of the CM programs considered (in terms of what the intervention involved), the target populations, and the tools used to measure the outcomes. This important drawback suggests that future clinical research needs a more detailed and clearly classified description of such interventions. At the same time, future secondary studies should clearly state their eligibility criteria in order to facilitate the interpretation of sources of variability. CM programs often differ in setting, case manager figure, elements of the program, and possibly also the components of single interventions. For example, the components of self-care support interventions can vary widely (Challis *et al*., 2010). As concerns the target population, a description of the tools used to enroll participants in CM schemes is essential to enable considerations on their external validity and generalizability (Smith *et al*., 2016). On this point, the main issues probably lie in the great variety of risk assessment tools used to assess the degree of a patientʼs multimorbidity (O’Caoimh *et al*., 2015), and the setting where this assessment is conducted will have an influence too. Finally, another problem concerns the different scales generally used to measure the same outcome in different studies, and the different periods of time considered: a longer-term relationship between patients and their case managers is likely to improve the success of a CM program (Hickam *et al*., 2013).

Studies should also provide details on the barriers to and facilitators of CM implementation across various primary care settings. For instance, one previous review (Kadu and Paul, 2015) found that the inner setting of the organization, the process of implementation, and characteristics of the individual healthcare providers could mediate the effectiveness of the primary care model.

Further investigations could focus on whether CM can be considered cost-effective or cost-saving from a societal and healthcare payment perspective by comparison with traditional fragmented practice. This could support healthcare decision-makers to conduct Health Technology Assessments that would also be applicable in the assessment of organizational models.

## Supplementary material

For supplementary material accompanying this paper visit http://dx.doi.org/10.1017/S1463423620000080. click here to view supplementary material
