# Supplementary material for: Health and health service usage outcomes of case management for patients with long-term conditions: a review of reviews
Source: Prim Health Care Res Dev. 2020 Aug 3;21:e26. doi: 10.1017/S1463423620000080 (PMC7443792; doi:10.1017/S1463423620000080)
Supplement: Supplementary file 1 [file S1463423620000080sup001.docx]

# Appendix

**Table I – Quality assessment**

| **Section/topic** | **Kumar, 2013** | **Huntley, 2013** | **Stokes, 2015** | **Hallberg, 2004** | **Oeseburg, 2009** | **Joo, 2017** | **Thomas, 2014** | **Joo, 2013** | **Latour, 2006** | **Kim, 2005** | **Hutt, 2004** | **You, 2013** | **Low, 2011** | **Lupari, 2011** | **Smith, 2016** | **Chiu, 2007** | **Eklund, 2009** | **Purdy, 2012** | **Boult, 2009** | **Soril, 2015** | **Hickam, 2013** | **Althaus, 2011** |
| --- | --- | --- | --- | --- | --- | --- | --- | --- | --- | --- | --- | --- | --- | --- | --- | --- | --- | --- | --- | --- | --- | --- |
|  | **SR** | **M** | **M** | **SR** | **SR** | **SR** | **SR** | **SR** | **SR** | **M** | **SR** | **SR** | **SR** | **SR** | **M** | **SR** | **SR** | **SR** | **SR** | **SR** | **SR** | **SR** |
|  | **C** | **A** | **A** | **C** | **B** | **B** | **B** | **C** | **B** | **A** | **C** | **A** | **A** | **C** | **A** | **C** | **B** | **A** | **C** | **A** | **A** | **A** |
| 1. **Title** | + | + | + | + | + | + | + | + | + | + | - | - | + | - | + | + | + | + | - | + | - | + |
| 1. **Structured summary** | + | + | + | - | + | + | - | + | + | + | + | + | + | + | + | + | - | + | - | + | + | + |
| 1. **Rationale** | + | + | + | + | + | + | + | + | + | + | + | + | + | + | + | + | + | + | + | + | + | + |
| 1. **Objectives** | + | + | + | + | + | + | - | - | + | + | + | + | + | + | + | - | + | + | - | + | + | + |
| 1. **Protocol and registration** | - | - | + | - | - | - | - | - | - | - | - | - | - | - | + | - | - | - | - | - | + | + |
| 1. **Eligibility criteria** | + | + | + | + | + | + | + | + | + | + | + | + | + | + | + | - | + | + | + | + | + | + |
| 1. **Information sources** | + | + | + | - | - | + | + | - | + | + | - | + | + | + | + | + | + | + | + | + | + | + |
| 1. **Search** | - | + | + | - | - | + | - | - | - | - | - | + | + | - | + | - | - | + | - | + | + | + |
| 1. **Study selection** | + | + | + | + | + | + | + | + | + | + | + | + | + | + | + | + | + | + | + | + | + | + |
| 1. **Data collection process** | + | + | + | - | + | + | + | - | + | + | - | + | + | + | + | - | + | + | - | + | + | + |
| 1. **Data items** | - | - | + | - | - | - | - | - | + | + | + | + | - | + | + | - | - | + | - | + | + | + |
| 1. **Risk of bias in individual studies** | - | + | + | - | + | + | + | - | + | + | + | + | + | - | + | - | + | + | + | + | + | + |
| 1. **Summary measures** | - | + | + | - | + | - | - | - | + | + | - | + | + | - | + | - | - | + | - | + | + | - |
| 1. **Synthesis of results** | - | + | + | - | + | - | + | - | - | + | - | + | + | - | + | - | - | + | - | + | + | - |
| 1. **Risk of bias across studies** | - | - | + | - | - | - | - | - | - | - | - | - | - | - | + | - | - | - | - | - | - | - |
| 1. **Additional analyses** | N/A | + | + | N/A | N/A | N/A | N/A | - | N/A | + | N/A | N/A | N/A | - | N/A | N/A | N/A | N/A | N/A | N/A | - | N/A |
| 1. **Study selection** | + | + | + | + | + | + | + | + | + | + | + | + | + | + | + | - | + | + | + | + | + | + |
| 1. **Study characteristics** | + | + | + | - | + | + | + | + | + | + | + | + | + | + | + | + | + | + | - | + | + | + |
| 1. **Risk of bias within studies** | + | + | + | - | + | + | + | - | + | + | - | + | + | - | + | + | + | + | - | + | + | + |
| 1. **Results of individual studies** | - | + | + | - | + | + | + | + | + | + | - | + | + | + | + | + | + | + | - | + | + | + |
| 1. **Synthesis of results** | + | + | + | + | - | + | + | + | + | + | + | + | + | - | + | + | + | + | + | + | + | + |
| 1. **Risk of bias across studies** | - | + | + | - | - | - | - | - | - | + | - | - | - | - | - | - | - | - | - | + | - | - |
| 1. **Additional analysis** | N/A | + | + | N/A | N/A | N/A | N/A | - | N/A | + | N/A | N/A | N/A | - | N/A | N/A | N/A | N/A | N/A | N/A | - | N/A |
| 1. **Summary of evidence** | + | + | + | - | - | + | + | - | + | + | + | + | + | + | + | + | + | + | - | + | + | + |
| 1. **Limitations** | + | + | + | - | + | + | + | - | + | + | - | + | + | + | + | - | + | + | + | + | + | + |
| 1. **Conclusions** | + | + | + | + | + | + | + | + | + | + | + | + | + | + | + | + | + | + | + | + | + | + |
| 1. **Funding** | - | + | + | - | - | + | - | + | - | - | - | + | + | + | + | + | + | + | + | + | + | + |

*SR = systematic review; M = meta-analysis*

*A = 80% or more; B = from 60% to 79%; C = 59% or less*
